# Dysregulation of the hypothalamic pituitary adrenal (HPA) axis and cognitive capability at older ages: individual participant meta-analysis of five cohorts

**DOI:** 10.1038/s41598-019-40566-x

**Published:** 2019-03-14

**Authors:** Michael Gardner, Stafford Lightman, Diana Kuh, Hannie Comijs, Dorly Deeg, John Gallacher, Marie-Claude Geoffroy, Mika Kivimaki, Meena Kumari, Chris Power, Rebecca Hardy, Marcus Richards, Yoav Ben-Shlomo

**Affiliations:** 1 Nuffield Department of Population Health, University of Oxford, Oxford, UK; 2 Henry Wellcome Laboratories for Integrative Neuroscience and Endocrinology, Bristol, UK; 3 MRC Unit for Lifelong Health and Ageing at UCL, London, UK; 4 Amsterdam Public Health Research Institute, VU University Medical Center, Amsterdam, The Netherlands; 5 Department of Psychiatry, University of Oxford, Oxford, UK; 6 McGill Group for Suicide Studies, McGill University, Montreal, Canada; 7 Department of Epidemiology and Public Health, University College London, London, UK; 8 ISER, University of Essex, Essex, UK; 9 Population, Policy and Practice, UCL, Great Ormond Street, Institute of Child Health, University College London, London, UK; 10 Department of Population Health Sciences, University of Bristol, Canynge Hall, Bristol, UK

## Abstract

Evidence on the association between functioning of the hypothalamic pituitary adrenal (HPA) axis and cognitive capability at older ages is mixed. We undertook a systematic review (until October 2016) and individual participant data (IPD) meta-analysis to test if dysregulation of the HPA axis is associated with worse cognitive capability. Five cohort studies were included in the IPD meta-analysis of diurnal cortisol patterns with crystallised and fluid cognitive ability. Higher night time cortisol was associated with worse fluid ability (standardised coefficient per SD increase −0.063, 95% CI −0.124, −0.002, *P* = 0.04; I^2^ = 79.9%; age and gender adjusted). A larger diurnal drop was associated with better fluid ability (standardised coefficient per SD increase 0.037, 95% CI 0.008, 0.065, *P* = 0.01; I^2^ = 49.2%; age and gender adjusted). A bigger cortisol awakening response (CAR) was weakly associated with better fluid (*P* = 0.09; I^2^ = 0.0%; age and gender adjusted) and crystallised (*P* = 0.10; I^2^ = 0.0%; age and gender adjusted) ability. There is weak evidence that a greater diurnal decline of the HPA axis and a larger CAR are associated with improvements in cognition at older ages. As associations are cross-sectional, we cannot rule out reverse causation.

## Introduction

Animal^1^ and human^2^ studies suggest that changes in the function of the hypothalamic pituitary adrenal (HPA) axis are related to functional ageing. HPA axis activates the secretion of glucocorticoids (cortisol in man and corticosterone in rodents) from the adrenal cortex, in response to stressful stimuli. Glucocorticoids exhibit a characteristic circadian rhythm. In humans, cortisol levels are typically high on waking, reach a peak at 30–45 minutes after waking and subsequently decline over the day, reaching a minimum near midnight^3^. Most studies focus on two dynamic measures of HPA activity^3^: (1) The size of the diurnal drop, that is the difference in the peak levels in the morning and nadir levels at night and (2) the cortisol awakening response (CAR) measured as the difference between the level on wakening and the level 30 minutes later.

In some rodent studies, aged animals have glucocorticoid levels showing a delayed return to normal levels following a stressful event^1^. This has been postulated to lead to a positive feedback loop, such that over time there are even greater glucocorticoid responses. Due to degenerative changes in the hippocampus, aged rats^4^ are impaired at inhibiting the secretion of glucocorticoids, following a stressful event. Degeneration is exacerbated by the cumulative exposure to glucorticoids and together these effects form the positive feedback loop. The so called ‘glucocorticoid cascade hypothesis’ has also been examined in humans. Such dysregulation of the HPA axis might lead to a flatter diurnal pattern in older individuals, as seen in the Whitehall II (WHII) study of British civil servants^5^.

Flatter diurnal patterns in older individuals have been shown to be associated with poorer cognitive capability^6^ as well as other adverse health outcomes such as cardiovascular disease^7^ and physical capability^8^. However, there is inconsistency in the results of studies assessing the association between cortisol levels and cognitive capability at older ages. Whilst some studies suggest that higher morning cortisol is associated with worse cognitive capability^9–11^, other studies have shown little evidence of this^12,13^. A flatter diurnal drop has been shown to be associated with poorer cognitive capability at older ages^6,14,15^, but many studies do not measure diurnal decline^10,13,16^.

Part of the inconsistency might be explained by the heterogeneity in the methods to measure cortisol; cortisol has been measured in urine^17,18^, serum^13,19^ and in saliva^6,20^. Furthermore, the outcome measures of cognitive capability in the literature are heterogeneous including verbal memory, verbal fluency and reasoning, executive function, processing speed and object recognition. Additionally, many of the studies use small unrepresentative samples^21,22^. To our knowledge, no systematic review of the literature has been undertaken to examine the association between cortisol levels and cognitive capability at older ages in larger population-representative cohorts.

Our study consists of two parts. First we undertook a systematic review and second, an individual participant data (IPD) meta-analysis^23^ of five cohort studies, as part of the Healthy Ageing across the Life Course (HALCyon) programme, to assess the association between measures of cortisol and cognitive capability. We classified cognitive capability into two summary measures: crystallized and fluid ability^24^. Crystallised intelligence has been defined as “a type of broad mental ability that develops through the “investment” of general intelligence into learning through education and experience^25^” and is relatively stable in ageing. Fluid intelligence has been defined as: “the ability to solve problems in unfamiliar domains using general reasoning methods^26^” and is more sensitive to age- and morbidity-associate decline”. Our approach has several advantages: (a) greater statistical power to detect modest associations; (b) standardising analyses by grouping cognitive capability and covariates in the same way across studies; (c) multiple measures of cortisol allowing a detailed characterisation of the association. We hypothesised that a lack of diurnal decline and hence higher cortisol levels across the day, reflecting dysregulation of the HPA axis, would be associated with worse cognitive capability.

## Results

**Figure 1:**
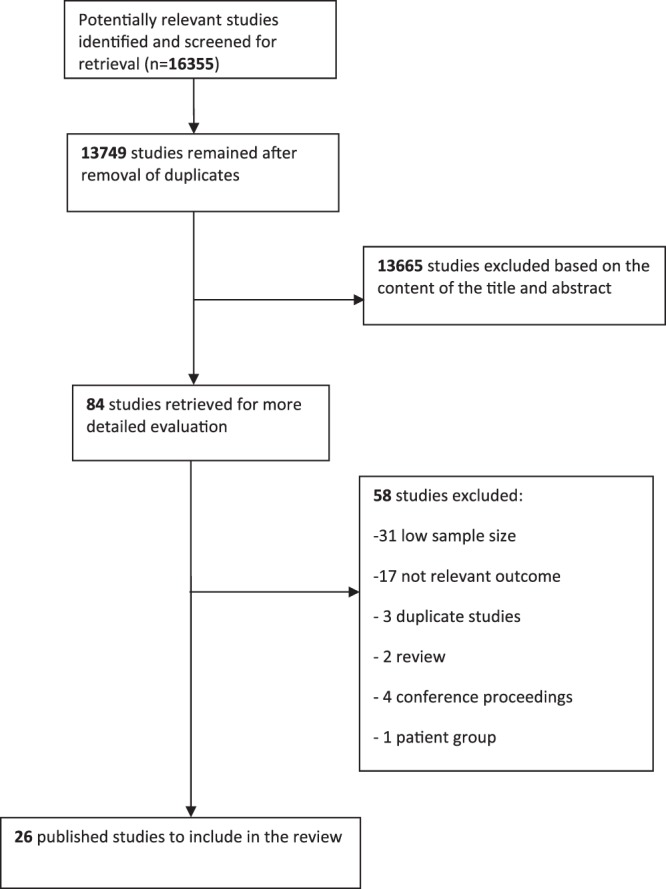
Flow diagram for identification of published studies for inclusion in review.

Our literature search identified 16,355 published studies (Fig. 1). Removing duplicate records left abstracts of 13,749 unique records to be screened. 13,665 studies were excluded based on the title and abstract and a further 58 studies were excluded following more detailed evaluation. Following these processes, 26 published studies were identified to be included in the review^6,9–20,27–39^. The characteristics of these studies are shown in Table 2. The sample sizes varied from 132 to 4,655 subjects. Cognitive capability was measured using a wide range of psychometric tests including composite screening tests, e.g. MMSE, and multiple specific tests e.g. verbal memory, with a high risk of a type I error due to multiple hypothesis testing. Most studies reported some association between a measure of HPA functioning and some cognitive outcomes but the actual measure varied between studies including higher morning cortisol, flatter diurnal drop, lower morning to evening ratio, bigger area under the curve and higher evening cortisol. In some cases, these measures were related to cognition only in sub-groups e.g. APOE-ε4 carriers. Since the outcome measures of cognitive capability across these 26 studies were heterogeneous, a formal meta-analysis of these studies was not justified (Table 2).

**Table 1:** Fluid ability measures.

Fluid ability	CaPS	LASA	NCDS	NSHD	Whitehall II
Verbal fluency (Animal Naming)	Participants name as many animals as possible in 60 seconds.		Participants name as many animals as possible in 60 seconds.		Participants name as many animals as possible in 60 seconds.
Verbal Memory		(a) AVLT is a test of verbal memory^10^, based on list learning. In LASA 15 words were learned in each of three trials and the learning score was the total number of words learned (maximum 45). (b) The Coding Task measures verbal associative memory^10^. Here, two rows paired characters are shown, each character in the upper row belonging to a character in the bottom row. The participant was then offered one character and had to verbally mention the missing paired second character. The participant had to complete as many character combinations as possible in one minute and the mean score for the three trials was used in the analyses.	(c) Immediate Verbal Memory by Immediate Memory recall task^11^. This was measured by how many words a participant could recall from a list of 10 common words immediately after the word list was read.	(a) AVLT is a test of verbal memory, based on list learning. In NSHD 15 words were learned in each of three trials and the learning score was the total number of words learned (maximum 45).	(a) AVLT is a test of verbal memory, based on list learning. In WHII participants were shown a list of 20 one or two-syllable words at 2- second intervals and asked to recall as many words in writing within 2 minutes.
Processing Speed (Letter cancellation task)			Letter Cancellation^11^ Participants were presented with a page containing 125 upper-case letters of the alphabet, of which 65 were target letters (P and W) and had to cross out as many target letters as possible in 1 minute.	Letter Cancellation^72^ Participants were presented with a page containing 125 upper-case letters of the alphabet, of which 65 were target letters (P and W) and had to cross out as many target letters as possible in 1 minute.	
Reaction Time	A choice reaction time task with a visual signal was used. Participants pressed one of four buttons as quickly as possible corresponding to which of the numbers 1 to 4 appeared in the signal screen.			A choice reaction time task with a visual signal was used. Participants pressed one of four buttons as quickly as possible corresponding to which of the numbers 1 to 4 appeared in the signal screen. Eight practice trials were given, followed by 40 real trials.	
Verbal and mathematical reasoning (Alice Heim Test)	Alice Heim test is made up of 65 items (33 mathematical reasoning and 32 verbal reasoning) of increasing difficulty. Participants identify patterns and infer principles and rules.				Alice Heim Test^73^ Made up of 65 items (33 mathematical reasoning and 32 verbal reasoning) of increasing difficulty. Participants identify patterns and infer principles and rules.
Non verbal reasoning (Raven’s Coloured Progressive Matrices- RCPM)		In RCPM^61^, the ability to deal with new information was measured by two subsets of 12 items (A and B). Each item consisted of drawing of a pattern with a section missing. Participants chose which of six patterns fitted the missing section. The items increased in difficulty and the maximum score was 24.

**Table 2:** Characteristics of studies included in the review.

Author	Cohort	Cortisol measure	Outcome Measure	Strongest Predictor (of worse cognition)
Alfaro *et al*.^16^	313 women and men 71–102 years	morning serum	MMSE	higher morning cortisol (women) cross-sectional
Beluche *et al*.^6^	197 women and men 65–90 years	saliva 3 times over day repeated next day	Verbal/visual memory,verbal fluency Cross-sectional and change	flatter diurnal drop longitudinal
Berteau-Pavy *et al*.^30^	116 women and men 62–92 years	saliva 8.30 am	Facial/Face/Object recognition Reaction time, Memory Island, MMSE	higher morning cortisol (men) cross-sectional
Comijs *et al*.^10^	1154 women and men 65–88 years	serum before 10 am	MMSE, AVLT, Coding Task Cross-sectional and change	higher morning cortisol cross-sectional
Fiocco *et al*.^32^	106 women and men 57.9 ± 0.40SE years	saliva 5 times over day	Declarative memory	No association
Fonda *et al*.^12^	1156 men 48–80 years	2 morning serum	Working memory, speed/attention spatial ability	No association
Franz *et al*.^33^	778 men 51–60 years	saliva 5 times over day repeated 3 separate days	General cognitive ability Neurocognitive battery including: Verbal memory, executive functioning	area under the curve cross-sectional
Gaysina *et al*.^20^	1796 women and men 60–64 years	saliva 4 times over day	Verbal memory Letter search speed, reaction time	higher evening cortisol cross-sectional
Geerlings *et al*.^36^	4244 women and men 44–45 years 76 ± 5SD years	morning and evening salivary cortisol	Memory, speed Executive functioning	higher evening cortisol cross-sectional
Geoffroy *et al*.^11^	4655 women and men	2 morning saliva	Verbal memory, verbal fluency Speed of processing	higher late morning cortisol longitudinal
Gerritsen *et al*.^15^	911 women and men 75.5 ± 6.8SD years	saliva 2 times over day	Global cognitive functioning Verbal memory, processing speed Baseline and at 4 years follow-up	flatter diurnal drop (APOE-ε4 carriers) longitudinal
Greendale *et al*.^19^	749 women 72.0 ± 8.1SD years	morning serum	Visual reproduction, MMSE Trails B, Category Fluency Cross-sectional and change	higher morning cortisol longitudinal
Johar *et al*.^37^	599 women and men 65–90 years	saliva 3 times over day	TICS-m with 4 domains: Orientation; memory; Attention/calculation and language	Lower morning to evening cortisol ratio (men) cross-sectional
Kalmijn *et al*.^28^	189 women and men 55–80 years	serum 8–9 am	MMSE Cross-sectional and change	Higher morning cortisol cross-sectional
Karlamanga *et al*.^18^	538 women and men 70–79 years	urinary 8 pm-8 am	Mental status questionnaire Cross-sectional and change	Higher overnight urinary cortisol longitudinal
Kuningas *et al*.^9^	563 women and men 85 years	serum before 11am	MMSE, speed, attention, recall Cross-sectional and change	Higher morning cortisol longitudinal
Lee *et al*.^31^	1140 women and men 50–70 years	saliva 4 times over day	Language, executive function Verbal/visual memory, speed	area under the curve cross-sectional
Mora *et al*.^35^	313 women and men 76.7 ± 7SD years	morning serum	MMSE Baseline and at 2 years follow-up	Higher morning cortisol (women) cross-sectional
O’ Hara *et al*.^14^	154 women and men 60–100 years	saliva 5 times over day	MMSE, speed, spatial verbal memory	flatter diurnal drop cross-sectional
Schrijvers *et al*.^13^	3341 women and men 72.0 ± 6.8SD years	morning serum	MMSE and Test Battery: Executive function, attention and Information processing speedInformation processing speed Baseline and 7 years mean follow-up	No association
Seeman *et al*.^17^	200 women and men 70–79 years	urinary 8 pm-8 am	Recall, spatial verbal memory Cross-sectional and change	Higher overnight urinary cortisol (women) longitudinal
Segerstrom *et al*.^38^	132 women and men 60–93 years	saliva 3 times over day	Verbal memory, executive function Cros-sectional and change	area under the curve longitudinal
Singh-Manoux *et al*.^27^	3229 women and men 61 years	saliva 6 times over day	Verbal memory, verbal fluency and Reasoning Baseline and 5 years mean follow-up	Flatter diurnal slope Higher night time cortisol in APOE-ε4 carriers longitudinal
Stawski *et al*.^34^	1500 women and men 33–84 years	saliva 4 times over day On 4 consecutive days	BTACT with fluid domains: Verbal memory, reasoning, working memory span, Executive functioning and processing speed	Higher night time cortisol cross-sectional
Stomby *et al*.^39^	200 women and men 55–80 years	saliva 4 times over day	Episodic memory, semantic memory, Visuospatial ability, working memory	No association
Wright *et al*.^29^	133 women and men 65–80 years	saliva 8 times over day	Declarative memory, matrix reasoning	cortisol response cross-sectional

Descriptive characteristics of the five studies included in the individual-participant data meta-analysis are shown in Table 3. Age range was from 50 to 88 years, the youngest cohort being NCDS (50.7 ± 0.15 SD years) and the oldest LASA (75.1 ± 6.4 SD years). There was some variation of the same crystallised and fluid cognition measures between studies, in part due to differences in test protocols. Mean NART totals were 28.0 ± 11.1 SD in CaPS and 35.9 ± 8.8 SD in NSHD. Verbal fluency ranged from 15.6 ± 3.8 SD in WHII to 22.6 ± 6.2 SD in NCDS. Verbal and mathematical reasoning was 26.3 ± 10.4 SD in CaPS and 44.2 ± 10.9 SD in the younger WHII cohort. Reaction time was 0.69 ± 0.20 SD seconds in CaPS and 0.62 ± 0.09 SD seconds in NSHD. Verbal memory (AVLT) ranged from 6.9 ± 2.4 SD in WHII to 24.8 ± 5.8 SD in NSHD, although there were variations in the tests. Processing speed was 265.5 ± 69.9 SD in NSHD and 335.8 ± 88.2 SD in NCDS.

**Table 3:** Characteristics of the participants aged 50–88 years, by study.

Variable	CaPS	LASA	NCDS	NSHD	Whitehall II
N	771	1151	4824	1165	2936
Gender (% male)	100	48.7	51.7	45.4	75.3
Age (years)	73.2 (4.0)	75.1 (6.4)	50.7 (0.15)	63	61.1 (5.9)
BMI (Kg/m^2^)	27.8 (3.9)	26.9 (4.2)	27.3 (4.8)	27.9 (4.8)	26.7 (4.3)
Current smoker (%)	13.6	18.1	17.9	16.5	7.8
Lower SEP (%)	60.4	39.9	33.4	25.2	53.5
Serum morning cortisol (nmol/L)	—	497.9 (170.8)	—	—	—
**Salivary cortisol (nmol/L)**					
T1 morning	19.6 (10.1)	—	21.2 (11.2)	23.4 (9.8)	20.0 (8.1)
T2	3.6 (5.5)	—	8.3 (7.1)	3.2 (3.4)	2.4 (2.7)
**Crystallised ability**					
NART	28.0 (11.1)	—	—	35.9 (8.8)	—
Mill Hill	—	—	—	—	25.1 (4.2)
GIT vocabulary test	—	12.9 (4.0)	—	—	—
**Fluid ability**					
Verbal fluency	17.7 (5.0)	—	22.6 (6.2)	—	15.6 (3.8)
**Verbal Memory**					
(i) *AVLT*	—	19.5 (6.2)	—	24.8 (5.8)	6.9 (2.4)
(ii) *Coding Task*	—	23.4 (7.1)	—	—	—
(iii) *Immediate memory*	—	—	6.7 (1.5)	—	—
Processing Speed	—	—	335.8 (88.2)	265.5 (69.9)	—
Reaction Time (s)	0.69 (0.20)	—	—	0.62 (0.09)	—
Verbal and mathematical reasoning	26.3 (10.4)	—	—	—	44.2 (10.9)
Non verbal reasoning	—	17.3 (4.5)	—	—	—

Results are presented as mean (SD), unless otherwise stated and are complete data including confounders and morning and night time cortisol measures (where available) and crystallised and fluid cognitive capability.

Serum cortisol level is a morning sample in LASA.

T1 salivary morning cortisol in CaPS, NSHD and Whitehall II was computed as the mean of the waking and 30 minute samples. In NCDS T1 was the 45 minutes after waking sample. In CaPS, NSHD and Whitehall II T2 was the night time cortisol sample and in NCDS it was the 3 hours 45 minutes after waking sample.

See methods for detailed descriptions of crystallised capability and fluid cognition measures. Crystallised capability is the National Adult Reading Test (NART) (0–50) in CaPS and NSHD, the Mill Hill Vocabulary Test (0–33) in Whitehall II and the GIT-vocabulary test in LASA. Fluid capability is derived by factor analysis of the fluid cognition measures in each of the cohorts. Fluid cognition measures in CaPS are animal naming, Alice Heim test (AH4) and reaction time (log_e_); Coding task, Auditory Verbal Learning Test (AVLT) or Verbal Memory and Ravens Coloured Progressive Matrices (RCPM) in LASA; Verbal memory (15 item word recall over 3 trials), search speed (0–600) and choice reaction time in NSHD and animal naming, verbal memory (20 item word recall) and AH4 in Whitehall II.

Across the cohorts, there was no standard method for classifying socioeconomic position. In CaPS, NCDS and NSHD, lower socioeconomic position was classified as manual (skilled manual, semi-skilled manual and unskilled) and higher socioeconomic position as non-manual (professional, managerial or skilled non-manual). In LASA, lower socioeconomic position was classified as low education level attained and higher socioeconomic position as middle and high education level attained. In Whitehall II, lower socioeconomic position was employment grade 1 and 2 and higher socioeconomic position was employment grade 3.

### Individual-participant data meta-analyses

**Figure 2:**
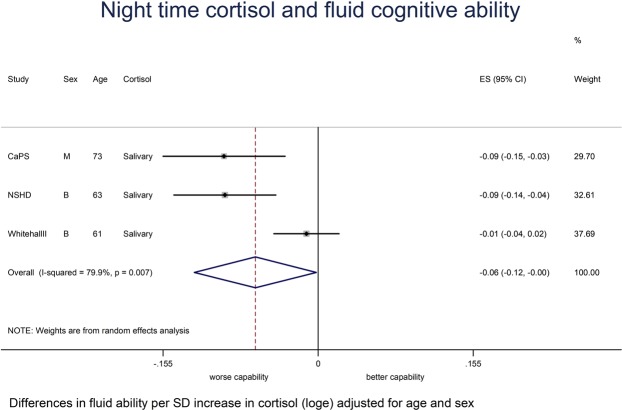
Meta-analysis for the association between night time cortisol and fluid cognitive ability adjusted for age and sex.

**Figure 3:**
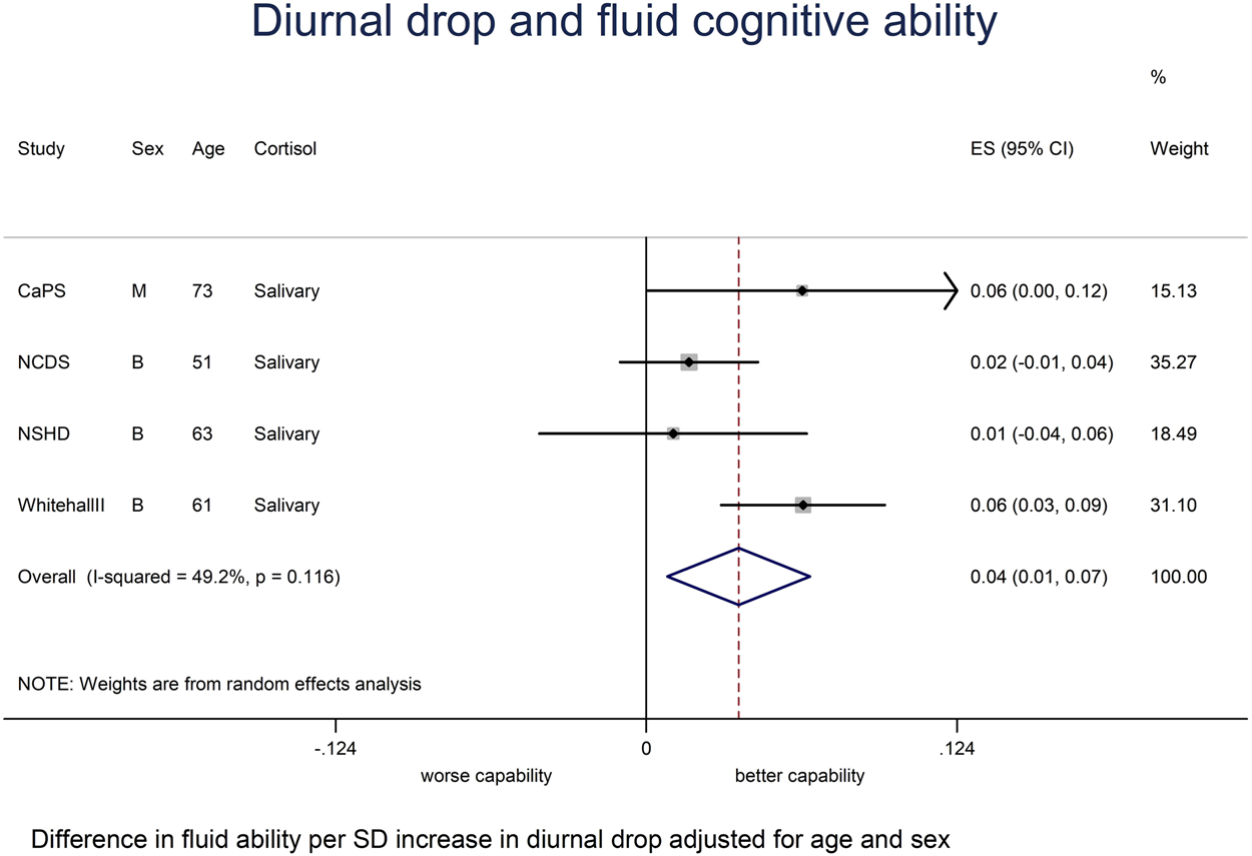
Meta-analysis for the association between diurnal drop and fluid cognitive ability adjusted for age and sex.

Total sample size in the age and sex-adjusted models varied between 5,131 and 12,143 depending on the meta-analysis (Table 4). Associations between the cortisol measures and crystallised and fluid ability from fixed or random effects meta-analyses were as follows: (a) Crystallised ability - bigger CAR (Table 4; Fig. S1d) was weakly associated with a better crystallised ability when age and gender adjusted (*P* = 0.10; I^2^ = 0.0%, *P* = 0.74) and this was little changed after further adjustments for BMI, smoking and socioeconomic position (*P* = 0.06; I^2^ = 0.0%, *P* = 0.93). No association was found between the other cortisol measures and crystallised ability (Table 4; Fig. S1). (b) Fluid ability - higher night time cortisol (Table 4; Fig. 2) was associated with a worse fluid ability (*P* = 0.04; I^2^ = 79.9%, *P* = 0.01) when age and gender adjusted, although this association was attenuated after adjusting for BMI, smoking and socioeconomic position (*P* = 0.10; I^2^ = 63.2%, *P* = 0.07). A larger diurnal drop (Table 4; Fig. 3) was associated with better fluid ability when age and gender adjusted (*P* = 0.01; I^2^ = 49.2%, *P* = 0.12), although this was again attenuated when further adjusting for BMI, smoking and socioeconomic position (*P* = 0.25; I^2^ = 61.3%, *P* = 0.05). A bigger CAR (Table 4; Fig. S2b) was weakly associated with a better fluid ability whether age and gender adjusted (*P* = 0.09; I^2^ = 0.0%, *P* = 0.43) or after additional adjustments for BMI, smoking and socioeconomic position (*P* = 0.07; I^2^ = 0.0%, *P* = 0.61).

**Table 4:** Overall summary estimates of effect for the associations between cortisol measures and cognitive capability from fixed or random effects meta-analyses.

Outcome and cortisol measure	Model A					Model B (Fully Adjusted)				
	(Age, sex adjusted)					(Age, sex, BMI, smoking status, socioeconomic position)				
	β^†^	95% CI	*P*-value	I^2^	*P-*value^‡^	β^†^	95% CI	*P*-value	I^2^	*P*-value^‡^
**Crystallised ability (sd score)**										
Morning^a^ (n = 6775)	−0.005	−0.050, 0.040	0.83	71.6%	0.01	−0.003	−0.034, 0.028	0.83	47.5%	0.13
Night time^b^ (n = 5285)	−0.021	−0.068, 0.026	0.39	65.3%	0.06	−0.007	−0.048, 0.035	0.75	59.6%	0.08
Diurnal drop^c^ (n = 5131)	0.021	−0.033, 0.076	0.44	72.8%	0.03	0.010	−0.024, 0.045	0.56	40.2%	0.19
CAR^d^ (n = 5159)	0.021	−0.004, 0.046	0.10	0.0%	0.74	0.023	−0.001, 0.047	0.06	0.0%	0.93
**Fluid ability (sd score)**										
Morning^a^ (n = 12143)	−0.008	−0.049, 0.032	0.69	80.0%	0.001	−0.007	−0.041, 0.027	0.68	73.1%	0.01
Night time^b^ (n = 5276)	−0.063	−0.124, −0.002	0.04	79.9%	0.01	−0.036	−0.080, 0.008	0.10	63.2%	0.07
Diurnal drop^c^ (n = 10497)	0.037	0.008, 0.065	0.01	49.2%	0.12	0.019	−0.013, 0.051	0.25	61.3%	0.05
CAR^d^ (n = 5136)	0.022	−0.003, 0.047	0.09	0.0%	0.43	0.022	−0.001, 0.046	0.07	0.0%	0.61

N = Sample size in age and sex adjusted analyses;† Differences in standardised crystallised ability; Differences in fluid ability; ‡ *P*-value is obtained from the heterogeneity χ²; ^a^Morning salivary cortisol is the average of the mean waking and 30 minutes post wakening samples in CaPS, NSHD and Whitehall II and in LASA, morning (before 10am) serum cortisol samples were taken; ^b^Night time cortisol in CaPS, NSHD and Whitehall II was transformed (log_e_); ^c^Diurnal drop is the difference between morning and night time salivary cortisol; ^d^CAR is the difference between the 30 min. post waking sample and the waking sample; All cortisol measures have been z-scored. Random effects meta-analyses were for I^2^ ≥40.2%, otherwise fixed effect meta-analyses were used.

### Heterogeneity and meta-regression analyses

There was evidence of heterogeneity between studies in age and sex-adjusted meta-analyses ranging from low (I^2^ = 0%, *P* = 0.43) for the associations between CAR and fluid ability to high (I^2^ = 80.0%, *P* = 0.001) for the associations between morning cortisol and fluid ability. In meta-regression analyses, there was no strong evidence that the associations between the various cortisol measures and either crystallised or fluid ability differed by age (above and below median), gender, BMI (obese or non-obese), smoking status (current smoker versus non-smoker) or socioeconomic position (higher versus lower socioeconomic position). There was weak evidence to suggest that the associations were stronger in obese than non-obese participants for night time cortisol and crystallised ability but this could have been due to chance (F = 4.64, *P* = 0.10). Furthermore, there was evidence to suggest that the associations were stronger in participants from lower versus higher socioeconomic position for diurnal drop and crystallised (F = 3.71, *P* = 0.13) or fluid ability (F = 12.60, *P* = 0.02). However, the former could have been due to chance and the latter due to a type 1 error (multiple testing).

### Sensitivity analyses

Re-running analyses using fixed-effect meta-analyses rather than random-effects meta-analyses had little effect on the associations (data not shown). We found little effect on the meta-analysis for the associations between diurnal drop and fluid ability when we omitted the NCDS cohort data. A larger diurnal drop was associated with better fluid ability when NCDS cohort was omitted (standardised coefficient per SD increase 0.048, 95% CI 0.016, 0.080, *P* < 0.01; age and gender adjusted), as we found when NCDS cohort was included (standardised coefficient per SD increase 0.037, 95% CI 0.008, 0.065, *P* = 0.01; age and gender adjusted).We found little effect on the meta-analyses for the associations between morning cortisol and i) crystallised ability (standardised coefficient per SD increase 0.008, 95% CI −0.042, 0.057, *P* = 0.74; age and gender adjusted) and ii) fluid ability (standardised coefficient per SD increase 0.05, 95% CI −0.037, 0.046, *P* = 0.83; age and gender adjusted) when we omitted the LASA cohort data. Re-running analyses using a Restricted Maximum Likelihood (REML) method rather than the DerSimonian-Laird random-effects method had little effect on the associations (data not shown).

## Discussion

Twenty six published observational studies were identified in our systematic review. Many of these found some association between cortisol and various cognitive outcomes, but since outcome measures of cognitive performance across these studies were heterogeneous, we did not carry out a meta-analysis of these studies. It is difficult to assess how much of the effects seen across these heterogeneous studies with some positive findings reflect a true causal association, or associations secondary to confounding, reverse causation, type I errors and publication bias. For our IPD meta-analysis, we found that higher night time cortisol was associated with worse fluid ability after adjustment for age and gender (high heterogeneity). A larger diurnal drop in cortisol was associated with better fluid ability (moderate heterogeneity). These associations were attenuated after adjustment for BMI, smoking and socioeconomic position. A larger CAR was weakly associated with better fluid (low heterogeneity) and crystallised capability (low heterogeneity), but no associations were found between morning cortisol, night time cortisol or diurnal drop and crystallised capability. We decided to adjust our associations for BMI as a measure of adiposity but whether adiposity is secondary to a less dynamic HPA axis^40^ or vice versa is unclear^41^. Our BMI-adjusted analyses might be over adjusted if BMI acts as an intermediary in the pathway between cortisol and cognitive capability.

Our findings are in agreement to some degree with the results of studies assessing the cross-sectional associations between cortisol levels and cognitive performance at older ages (Table 2). Higher night time cortisol has been shown to be associated with poorer cognitive performance at older ages^34,36^, although other studies have found little evidence of such an association^39^. A larger diurnal drop has been shown to be associated with better cognitive performance at older ages^6,15^, although many studies did not measure diurnal decline (Table 2). Our results showed that a larger CAR was weakly associated with better fluid and crystallised capability. In the Vietnam Era Twin Study of Aging (VETSA)^33^, a larger CAR was associated with poorer cognitive performance, although this association was attenuated after adjusting for area under the curve (AUC). Many studies however, did not measure CAR (Table 2). Our results showed little evidence of an association between morning cortisol and cognitive performance, in agreement with some of the literature^12,13^; however, several studies have shown that higher morning cortisol is associated with worse cognitive performance (e.g.^11,35^; Fig. S1a; LASA cohort).

There are several potential explanations for the inconsistencies in the literature. First, cortisol has been measured using average cortisol measures such as urinary cortisol^3^, or has been measured only in a morning serum sample. Second, many of the studies in the literature use small unrepresentative samples e.g.^21,42^. Third, the outcome measures of cognitive performance in the literature are heterogeneous and include verbal memory and verbal fluency^6^, executive function^33^, processing speed^15^ and object recognition^30^. Hence we classified cognitive performance into crystallized intelligence (stable in maturity and representing the investment of general intelligence into skills, knowledge, and experience) and fluid intelligence (more vulnerable to age- and morbidity-associated decline, and concerned with reasoning and on the spot problem solving in novel situations^24^). We note however, that heterogeneity in our IPD meta-analyses ranged from low to high. Fourth, cortisol is secreted in a pulsatile manner^43^. This means that single samples are not representative of average levels at any particular time and multiple samples would be needed to really understand cortisol concentrations at any one time. Frequently therefore there is considerable measurement error in characterising the HPA axis and for CAR for instance, it has been recommended that CAR be performed at least twice on two separate days^44^.

Several longitudinal studies have investigated the association between cortisol and cognitive performance (Table 2). Higher urinary cortisol measures at baseline have been associated with poorer cognitive performance at follow-up^17,18^. Studies of the association between morning serum cortisol at baseline and cognitive performance at follow-up are contradictory (Table 2). These longitudinal studies did not have measures of diurnal cortisol profiles and in the Whitehall II study^27^, there was little evidence of a longitudinal association between diurnal cortisol patterns and cognitive performance. This cohort study of civil servants does not include blue collar workers or unemployed people limiting its generalisability. In the Longitudinal Aging Study Amsterdam (LASA)^15^, lower morning cortisol, higher night time cortisol levels and flatter diurnal slope, were associated with increased risk of memory decline in APOE-ɛ4 carriers but not in non-carriers, and this sub-group analysis needs replication. Inconsistent associations are elegantly demonstrated in a study of community dwelling older participants^6^, which found that a flatter diurnal slope was associated with decline in visuo-spatial performance and visual memory in men but in verbal fluency in women. In one small cohort study, higher event-based stress was associated with faster cognitive decline^45^ only in subjects with cognitive impairement at baseline and paradoxically higher mean daily cortisol in this sub-group was associated with slower decline.

One approach to examine the HPA dysfunction hypothesis is to look at cognition in patients with Cushing’s syndrome, who have chronic exposure to elevated levels of cortisol. This syndrome has been associated with deficits in several areas of cognitive performance, including non-verbal memory and visual and spatial information^46^. For both patients with Cushing’s syndrome^47^ and in older adults^48^, exposure to high cortisol levels has been shown to be associated with a smaller volume of the hippocampus. Such findings might subsequently lead to hippocampal atrophy^1^. However, in a recent study^39^, higher area under the daytime cortisol curve (AUC- average cortisol across the day) was not associated with hippocampal volume, but was inversely associated with prefrontal cortical surface area and with prefrontal cortical thickness.

The key strength of this pooled analysis is that it is based on five adult cohort studies with a large combined sample size (ranging from n = 5,131 to 12,143 participants). We undertook a 2-step IPD meta-analysis^23^ which provided greater statistical power to detect modest associations and enabled us to standardise analyses by grouping cognitive performance and covariates in the same way across studies. We classified cognitive performance into crystallized and fluid capability to reduce multiple testing and enable standardization across cohorts. Even with this approach, there was statistical evidence of between studies heterogeneity (I^2^ varying between 49.2% to 80%) and we did not find many factors which explained this heterogeneity, although we were probably underpowered for this. Alternatively to increase the power of the analyses, multivariate meta-analyses^49^ might be undertaken in future studies. Whilst we observed some heterogeneity of effects with the CAR measures, we acknowledge that the outcomes we derived within each study may violate the assumption of measurement invariance and this may introduce artefactual heterogeneity. Our measures of the HPA axis will have measurement error, particularly for those studies using a single serum cortisol measure (i.e. LASA) and measures made on multiple days are recommended^44^. In line with current guidelines^50^, we recommend that future studies on CAR use objective methods for verification of awakening times, such as polysomnography or wrist actigraphy. We could not exclude reverse causation, given our cross-sectional data. This is highlighted in the Vietnam Era Twin Study of Aging^33^ where worse cognitive performance at age 20 predicted elevated midlife cortisol and a smaller diurnal drop in midlife, and also by previous research on the NCDS suggesting that cortisol-cognitive performance associations at older ages may be due, in part, to associations from earlier in life i.e. with childhood cognition^11^. Our selection of cohorts may also be criticised as they spanned a range of birth cohorts that were predominantly from the United Kingdom which limits their generalizability.

In conclusion, we did not find any evidence to suggest strong associations between HPA dysfunction and worse cognition, but there was some evidence that a more responsive HPA axis is associated with better cognitive performance in later life. However, these are modest associations and, furthermore, are cross-sectional in nature so reverse causation cannot be ruled out. We would recommend that future studies either use some form of multiple sampling technology or at least collect both morning and night time samples, for several days, and look into associations between change in cortisol and change in cognitive performance to help untangle causality.

## Methods

We undertook a systematic review of the published literature following the meta-analysis of observational studies in epidemiology (MOOSE) guidelines^51^ and the PRISMA statement^52^.

### Selection criteria

Eligible observational studies were those conducted on individual participants that examined the association between measures of diurnal cortisol patterns and cognitive capability. Eligible study populations were community dwelling older adults, identified in titles and/ or abstracts. Eligible studies had to have a minimum number of 100 participants and we excluded studies of patient or disease-selected groups e.g. diabetic patients.

### Literature search and additional studies

Searches of the electronic databases MEDLINE and EMBASE (from 1950 or 1980 up to 25^th^ October 2016) were performed using text word search terms and explosion MeSH terms (Supplementary Methods) by MG. Initially we had aimed to undertake a data extraction from the systematic review of the literature and combine the results with the individual participant data from the HALCyon cohorts. Since the outcome measures of cognitive capability across these 26 studies were heterogeneous, a formal meta-analysis of these studies was not justified. We did not undertake a data extraction, or assess the quality of each study, but we did summarise the characteristics of these studies.

### The cohorts

The HALCyon research programme on cortisol and ageing outcomes involves nine UK cohort studies. Three of these cohorts have data on both cortisol and cognitive capability: the Caerphilly Prospective Study (CaPS)^53^; the 1958 British Birth Cohort (NCDS)^11,54^; the MRC National Survey of Health and Development^20,55^. We have additionally included two large-scale cohort studies identified through the HALCyon collaboration: the Longitudinal Ageing Study Amsterdam (LASA)^15,56^ and the Whitehall II (WHII) study^27,57^. Details of the cohorts are given in Supplementary Methods.

### Cortisol measures

In LASA, morning (before 10am) serum cortisol samples were taken in cycle 2 (age 64–88 years) and the serum levels were determined using a competitive immunoassay^10^. The inter-assay and intra-assay coefficients of variation were below 8% and 3% respectively.

Salivary cortisol samples were collected in CaPS (age 65–83 years), NCDS (age 44–45 years), NSHD (age 60–64 years) and WHII (age 50–73 years). Participants were shown how to collect saliva using plain cotton wool swabs (salivettes) at home. Participants were asked to chew on the salivettes for one to two minutes and a saliva sample was obtained. In CaPS, participants took samples on waking, 30 minutes after waking, at 2 pm and at 10 pm over two consecutive days. In NCDS, samples were taken 45 minutes after waking (T1) and 3 hours later (T2). In NSHD samples were taken on waking, 30 minutes after waking and at 9 pm. A mid-morning sample was also taken in NSHD at the clinic visit but has not been used in this analysis. In WHII, samples were taken on waking, 30 minutes after waking and at waking +2.5 h, +8 h, + 12 h and at bedtime. Samples were frozen and subsequently assayed by chemiluminescence. In CaPS, NSHD and WHII morning salivary cortisol was computed as the mean of waking and 30 minute samples. In NCDS, the 45 minutes after waking sample was used as the morning salivary cortisol sample (T1). In each of CaPS, NCDS, NSHD and WHII assays were done in the same laboratory (Dresden) specialising in high through-put cortisol assays^58^. In CaPS, the inter-assay coefficient of variation for the salivary cortisols was 4% at both low (5.3nmol/l) and high controls (39.0nmol/l)^59^. The inter-assay and intra-assay coefficients of variation were less than 10% in NCDS^11^, less than 6% in NSHD and less than 8% in WHII^5^.

### Cognitive capability measures

Crystallised and fluid ability measures were taken in CaPS (age 65–83 years), LASA (age 64–88 years), NCDS (age 50 years) and WHII (age 50–73 years). In NSHD, crystallised ability measures were taken at age 53 and fluid ability measures were taken at age 62–65 years.

#### Crystallised ability

In CaPS and NSHD the crystallised capability measure was the National Adult Reading Test (NART)^60^, a word pronunciation test with maximum score 50, highly correlated with general cognitive ability. In Whitehall II, the Mill Hill Vocabulary test^61^ encompassed the ability to recognise and comprehend words, consisting of a list of 33 stimulus words of increasing difficulty and six response choices per word. In LASA, crystallised intelligence was measured by the Groninger Intelligentie Test (GIT)^62^. Here, 20 words of increasing difficulty are presented and the participant chooses a synonym from five alternatives and the maximum score is 20. In NSHD, the NART score was taken at age 53, which is the only cohort where the crystallised capability measure predated the cortisol measure.

#### Fluid ability

The fluid ability measures for each cohort are detailed in Table 1. We derived one fluid capability measure per cohort and these details are given in the statistical analyses section.

### Clinical and questionnaire-based data

Anthropometric measures were taken at clinic (CaPS, NSHD and WHII) or by medical interview at home (NCDS and LASA). Standard height was measured to the nearest mm using a stadiometer. Weight was measured in Kg using standardised scales (CaPS and NSHD), a SECA floor scale (LASA), Tanita solar scales (NCDS) or by an electronic Soehule scale (Leifheit AS) with a digital readout (WHII). Body Mass Index (BMI) was calculated as weight divided by height^2^ (Kg/m^2^). Smoking behaviour was assessed by self-completed questionnaire (CaPS, NCDS and WHII) or medical interview (LASA and NSHD). The derived variable for smoking status was classified into never, past or current. Across the cohorts there was no standard method for classifying socioeconomic position (SEP). In CaPS, NCDS and NSHD, SEP was defined by the British Registrar General’s classification of occupation and based on own occupation in adult life. The grouping was I or II (professional/managerial), IIINM (skilled non-manual), IIIM (skilled manual) and IV and V (semi-skilled and unskilled manual). In CaPS SEP was measured at phase 2, in NCDS at age 42 years and in NSHD at age 53. Lower SEP was classified as manual and higher SEP as non-manual. In LASA, SEP was defined according to education level attained^62^. Lower SEP was classified as low education level attained (elementary not completed and elementary education) and higher SEP as middle (including intermediate vocational education and general secondary education) and high (including college education and university) education level attained. In WHII, SEP at phase 7 was defined according to last known employment grade, where lower SEP was employment grade 1 and 2 and higher SEP was employment grade 3.

### Statistical analyses

Since cortisol has a marked circadian rhythm, we adjusted for times of sampling in CaPS, NCDS, NSHD and WHII, the cohorts with measures of salivary cortisol. In CaPS, NSHD and WHII, data on the actual times at which salivary cortisol samples were taken were available. In CaPS, NSHD and WHII we fitted a linear or polynomial function to the association between cortisol and time of measurement and when time of sampling predicted cortisol levels, we added residuals from the best fit model to the overall mean cortisol value^53^. To take into account time of sampling in NCDS, cortisol values for each individual were centred at 45 minutes after the mean waking time and at 3 hours 45 minutes after the mean waking time^11^. In addition to the morning (CaPS, LASA, NCDS, NSHD and WHII) and night time (CaPS, NSHD and WHII) samples, we derived the diurnal drop in CaPS, NCDS, NSHD and WHII. In CaPS, NSHD and WHII this was the difference between the morning and evening salivary cortisol samples. In NCDS, the diurnal drop was (T1 − T2)/3. We z-scored each of the cortisol measures including morning cortisol and night time cortisol, as well as the derived diurnal drop measure. In CaPS, NSHD and WHII, we also calculated the ‘Cortisol Awakening Response’ (CAR) (difference between the 30 minutes post waking sample and the waking sample). We also z-scored the derived CAR measure. We excluded participants treated by oral corticosteroid medication and in WHII we excluded participants who took samples later than 10 minutes after waking.

Whilst free cortisol concentrations are found in saliva, in serum cortisol levels represent total protein bound and free cortisol concentrations. In a study investigating the association between serum and salivary cortisol levels in healthy individuals^63^, correlations were high. We converted absolute cortisol levels to study- specific z-scores (mean 0 and standard deviation 1). Night time cortisol was positively skewed and was therefore transformed (log_e_). We also converted the transformed night time cortisol, diurnal drop and CAR to study- specific z-scores.

The crystallised ability measures (GIT, Mill Hill Vocabulary test and NART) were standardised by computing study-specific z-scores to take into account protocol variability. For each cohort, fluid ability was derived by performing factor analysis on the three fluid cognition measures in CaPS (Animal naming, AH4 and reaction time), LASA (Coding Task, RCPM and verbal memory), NCDS (Animal naming, letter cancellation and immediate memory), NSHD (Reaction time, verbal memory and letter cancellation) and WHII (Animal naming, AH4 and verbal memory). The factor analysis resulted in standardised fluid ability outcome measures (mean 0 and standard deviation 1). In the factor analysis we specified that the principle component factor method be used so that communalities are assumed to be 1.

We used linear regression models to analyse crystallised ability and fluid ability. We chose potential confounders for the analysis from the literature (age^64^, sex^64^, adiposity^64^, smoking status^65^ and socioeconomic position^66^). We adjusted the final multivariable model for age, sex, body mass index (BMI) (Kg/m^2^), smoking status (never, past or current) and socioeconomic position (higher, lower).

We undertook a two stage meta-analysis of individual participant data with each model initially run within each cohort (the first stage) for CaPS, LASA, NSHD and WHII. The cohort- specific effect estimates and standard errors were then pooled by running random-effects meta-analysis using the DerSimonian and Laird method^67^. For the second stage, the co-authors from NCDS completed a standardised table with specific effect estimates and standard errors. We initially adjusted these analyses for age and sex and then additionally for BMI, smoking status and SEP as potential covariates that may confound the association. We investigated between study heterogeneity using I^2^ statistic^68^. We examined potential sources of heterogeneity for age (above versus below median age), sex, adiposity (BMI at ≥30Kg/m^2^ versus <30Kg/m^2^ for obese and non-obese participants), smoking status (current smoker versus non-smoker) and SEP (higher versus lower SEP) by stratifying random-effects meta-analyses by each of these factors and by running meta-regression analyses^69^. For meta-regression analyses, we used post-estimation Wald tests to obtain F ratios and p values.

### Sensitivity analysis

We ran a fixed-effect meta-analysis using the Mantel-Haenszel method^70^ and compared the results with the random-effects meta-analysis. Due to differences in the methodology for calculating diurnal drop in NCDS, we repeated the meta-analysis for the associations between diurnal drop and fluid ability, but omitting the NCDS cohort data. Due to the difference of the measurements of cortisol in LASA (serum cortisol in LASA and salivary cortisol in the other cohorts), we repeated the meta-analyses for the associations between morning cortisol and i) crystallised ability and ii) fluid ability, but omitting the LASA cohort data. Since the DerSimonian-Laird estimator may underestimate the between-study heterogeneity^71^, we ran a Restricted Maximum Likelihood (REML) method and compared the results with the DerSimonian-Laird random-effects method.

## Supplementary information

Supplemental information

## Data Availability

All requests for collaboration on the Caerphilly Prospective Study are reviewed by an independent steering committee (http://www.bris.ac.uk/social-community-medicine/projects/caerphilly/collaboration/). MRC National Survey of Health and Development data used in this publication are available to bona fide researchers upon request to the NSHD Data Sharing Committee via a standard application procedure. Further details can be found at http://www.nshd.mrc.ac.uk/data. doi: 10.5522/NSHD/Q101; doi:10.5522/NSHD/Q102. National Child Development Study data are available via registration with the UK Data Service. Data from the Longitudinal Aging Study Amsterdam (LASA) are available for use for specific research questions, provided that an agreement is made up. Research proposals should be submitted to the LASA Steering Group, using a standard analysis proposal form that can be obtained from the LASA website: www.lasa-vu.nl. Files with data published in this publication are freely available for replication purposes and can be obtained using the same analysis proposal form. The LASA Steering Group will review all requests for data to ensure that proposals for the use of LASA data do not violate privacy regulations and are in keeping with informed consent that is provided by all LASA participants. Whitehall II data, protocols, and other metadata are available to the scientific community. Please refer to the Whitehall II data sharing policy at https://www.ucl.ac.uk/whitehallII/data-sharing.
