# Supplementary material for: Dysregulation of the hypothalamic pituitary adrenal (HPA) axis and cognitive capability at older ages: individual participant meta-analysis of five cohorts
Source: Sci Rep. 2019 Mar 14;9:4555. doi: 10.1038/s41598-019-40566-x (PMC6418174; doi:10.1038/s41598-019-40566-x)
Supplement: Supplementary file 1 — Supplemental information [file 41598_2019_40566_MOESM1_ESM.docx]

**Supplementary Information**

**Dysregulation of the hypothalamic pituitary adrenal (HPA) axis and cognitive capability at older ages: individual participant meta-analysis of five cohorts.**

Michael Gardner^1,^*, Stafford Lightman^2^, Diana Kuh^3^, Hannie Comijs^4^, Dorly Deeg^4^, John Gallacher^5^, Marie-Claude Geoffroy^6^, Mika Kivimaki^7^, Meena Kumari^7,8^, Chris Power^9^, Rebecca Hardy^3^, Marcus Richards^3^ & Yoav Ben-Shlomo^10^

^1^Nuffield Department of Population Health, University of Oxford, UK. ^2^Henry Wellcome Laboratories for Integrative Neuroscience and Endocrinology, Bristol, UK. ^3^MRC Unit for Lifelong Health and Ageing at UCL, London, UK. ^4^Amsterdam Public Health Research Institute, VU University Medical Center, Amsterdam, the Netherlands. ^5^Department of Psychiatry, University of Oxford, UK. ^6^ McGill Group for Suicide Studies, McGill University, Canada. ^7^Department of Epidemiology and Public Health, University College London, UK. ^8^ISER, University of Essex, UK. ^9^Population, Policy and Practice, UCL, Great Ormond Street, Institute of Child Health, University College London, UK. ^10^ Department of Population Health Sciences, University of Bristol, Canynge Hall, Bristol, UK.

**Supplementary Methods**

***Searches of electronic databases MEDLINE and EMBASE (1950 or 1980 to 25^th^ October 2016) showing MeSH terms and text word search terms.***

The following electronic databases were searched to identify potentially eligible published papers, letters, abstracts and review articles: MEDLINE (from 1950 to 25/10/2016); EMBASE (from 1980 to 25/10/2016). These searches were conducted using the following search terms: **explosion** **Mesh terms;** Hydrocortisone/; Receptors, Glucocorticoid/; Hypothalamo-Hypophyseal System/; Pituitary-Adrenal System/; Cognition/; Neuropsychological Tests/; Memory/; Verbal Learning/; Psychomotor Performance/; Psychometrics/; Reaction Time/; Intelligence Tests/. **Free text search terms;** cortis$.tw; hydrocort$.tw; glucocorti$ receptor.tw; HPA.tw; Hypothalam$ Pituitary Adrenal axis.tw; cognit$.tw; cognitive function.tw; cognitive performance.tw; verbal learning.tw; verbal fluency.tw; memory performance.tw; memory function.tw.

***Cohort details for CaPS, LASA, NCDS, NSHD and Whitehall II.***

*Caerphilly Prospective Study (CaPS)*

CaPS is a cohort of men born between 1920 and 1939 who were recruited between 1979 and 1983 from Caerphilly and adjacent villages. At ages 45-59 years, 2512 men (response rate of 89%) were seen and followed up at phase 2 (1984-1988), phase 3 (1989-1993), phase 4 (1993-1996) and phase 5 (2002-2004). An additional 447 men of similar age were recruited at phase 2 who had moved into the defined area. Cortisol and cognitive capability measures undertaken at phase 5 are those included in the current study.

*Longitudinal Ageing Study Amsterdam (LASA)*

The Longitudinal Ageing Study Amsterdam (LASA) is a cohort study on predictors and consequences of changes in cognitive, emotional, physical and social functioning in older persons. In 1992-1993, at ages 55-85 years, baseline measurements were undertaken and participants were followed up at cycle 2 (1995-1996), cycle 3 (1998-1999), cycle 4 (2001-2002), cycle 5 (2005-2006) and cycle 6 (2008-2009). Blood samples from which cortisol could be determined were available for 1273 participants and cycle 2. Serum was stored at -70^0^C until processing in 2002/2003. Cortisol and cognitive capability measures undertaken at cycle 2 are those included in the current study.

*1958 British Birth Cohort (National Child Development Study- NCDS)*

NCDS is the 1958 British Birth Cohort is a population cohort consisting of 17,638 participants enrolled in the Perinatal Mortality Survey born in 1 week in March 1958 in England, Scotland and Wales and 920 immigrants with the same birth dates included to age 16 years. Information has since been collected at ages 7, 11, 16, 23, 33, 42, 45 and 50 years. At 44-45 years participants underwent a biomedical examination and saliva samples for cortisol assessment were collected. Cognitive capability measures were undertaken when participants were aged 50 years.

*MRC National Survey of Health and Development (NSHD)*

NSHD is the 1946 British Birth Cohort and is a representative sample of people born in England, Scotland and Wales during one week in March 1946 and have been followed up prospectively since birth. 1880 participants had saliva collected when they were aged 60-64 years and processed in 2009/2011. Cognitive capability measures were undertaken when participants were aged 62-65 years.

*Whitehall II Study*

Whitehall II is a cohort of men and women initially recruited between 1985 and 1988 (phase 1) from 20 London-based civil service departments when participants ages ranged from 35-55 years. 10,308 people participated at phase 1 and they have been followed up since and eight phases have been completed. At phase 7 (2002-2004), 6484 participants had a clinical assessment and cortisol and cognitive capability measures undertaken and are those included in the current study.

**Supplementary Figures**

**Figure S1** Meta-analysis for the associations between morning cortisol and crystallised cognitive ability (a), night time cortisol and crystallised cognitive ability (b), diurnal drop and crystallised cognitive ability (c) and PACR (post-awakening cortisol levels) and crystallised cognitive ability (d) adjusted for age and sex

**Figure S2** Meta-analysis for the association between morning cortisol and fluid cognitive ability (a) adjusted for age and sex and PACR (post-awakening cortisol levels) and fluid cognitive ability (b) adjusted for age and sex

**Figure S1a**

**Figure S1b**

**Figure S1c**

**Figure S1d**

**Figure S2a**

**Figure S2b**
